# Supplementary figures and images for: Beyond identity: Understanding the contribution of the 5’ nucleotide of the antisense strand to RNAi activity
Source: PLoS One. 2021 Sep 7;16(9):e0256863. doi: 10.1371/journal.pone.0256863 (PMC8423273; doi:10.1371/journal.pone.0256863)

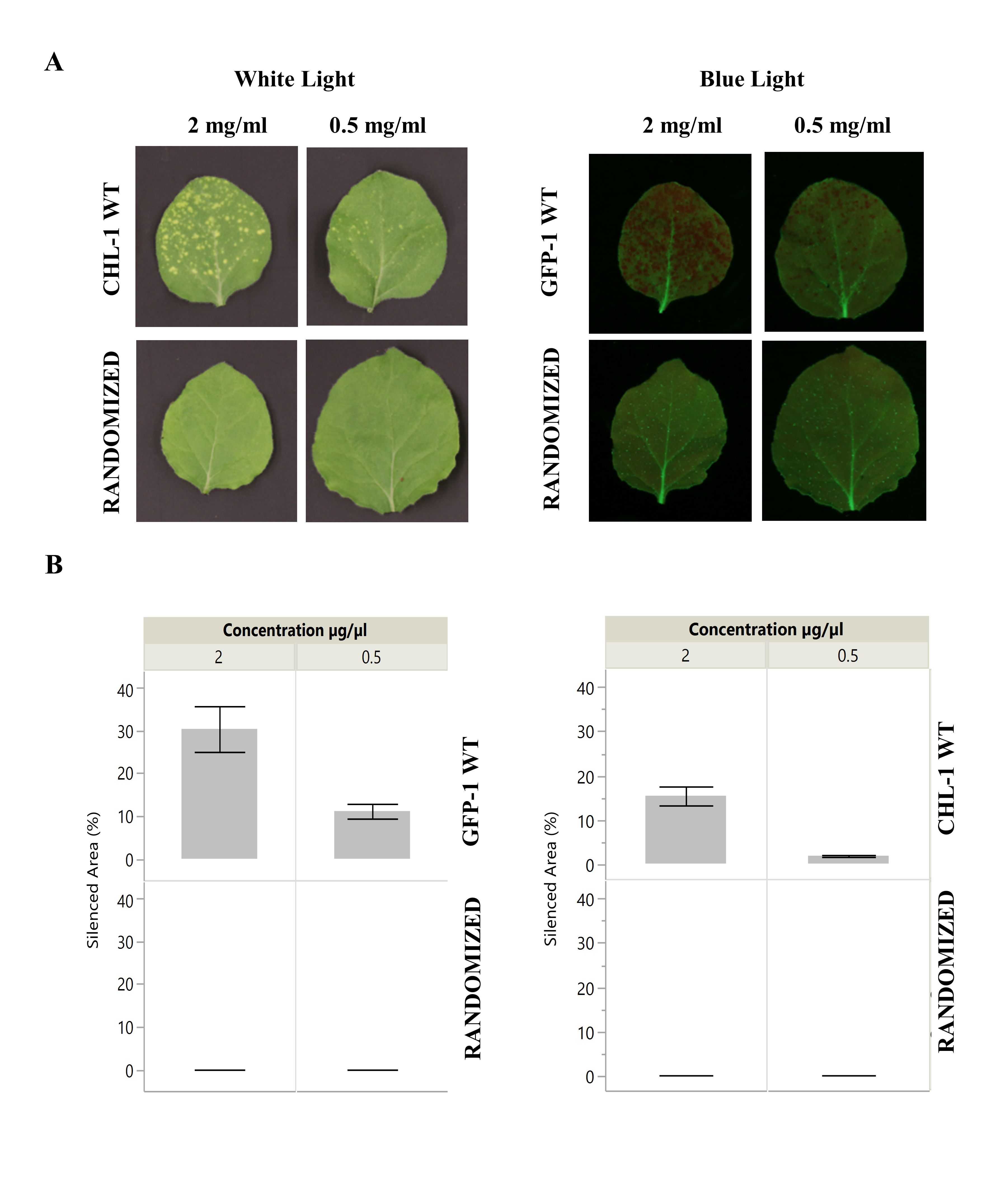

Supplement: S1 Fig — A, Leaves were treated with the indicated triggers at 2 concentrations by sandpaper abrasion and were photographed 4 dpa under white or blue light. B, Phenotypic area was quantified with Image J and is graphed as percent of the treated leaf area; error bars represent standard error of the means. (TIF) [file pone.0256863.s001.tif]

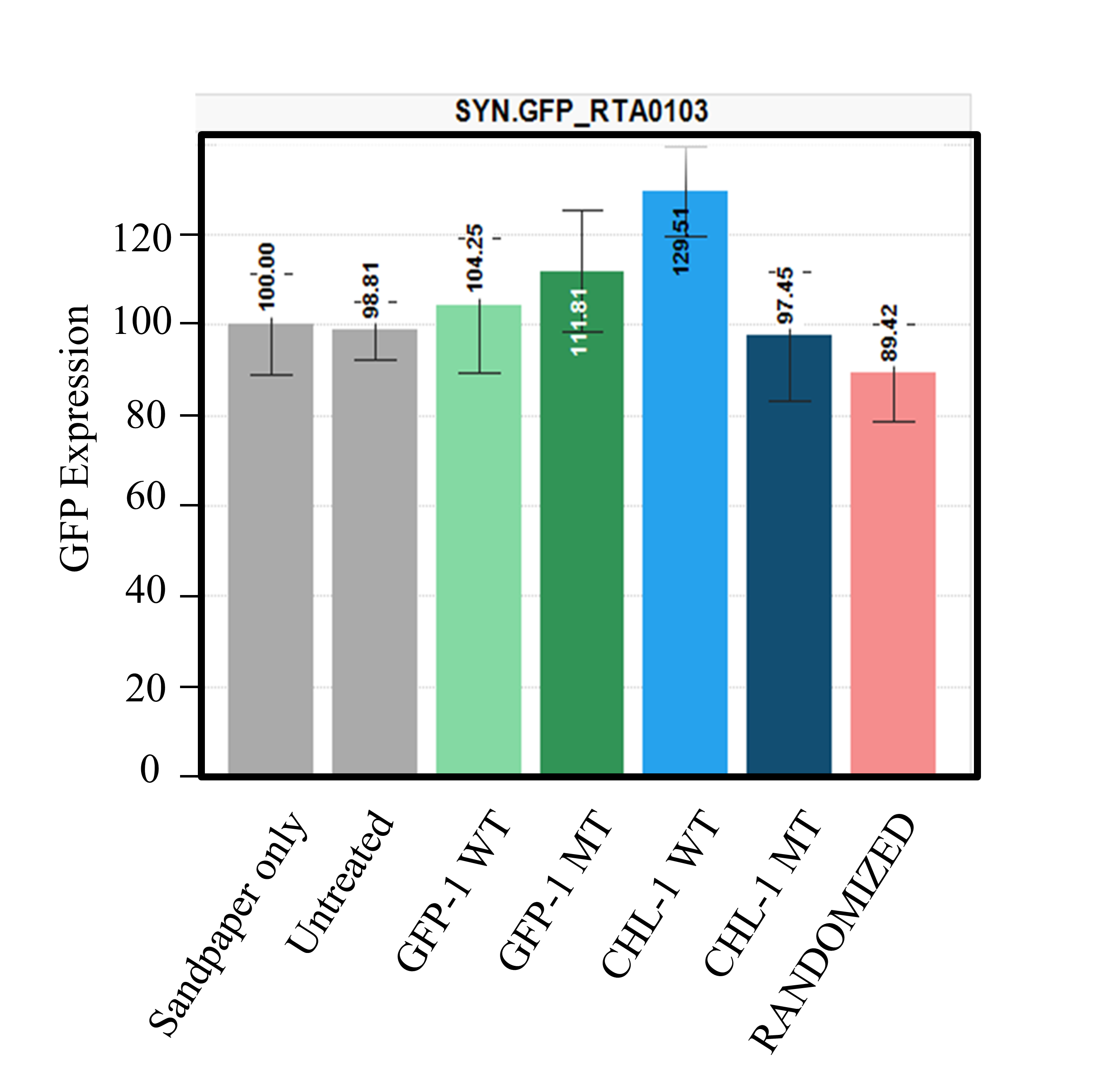

Supplement: S2 Fig — The error bars represent standard error of the means. (TIF) [file pone.0256863.s002.tif]

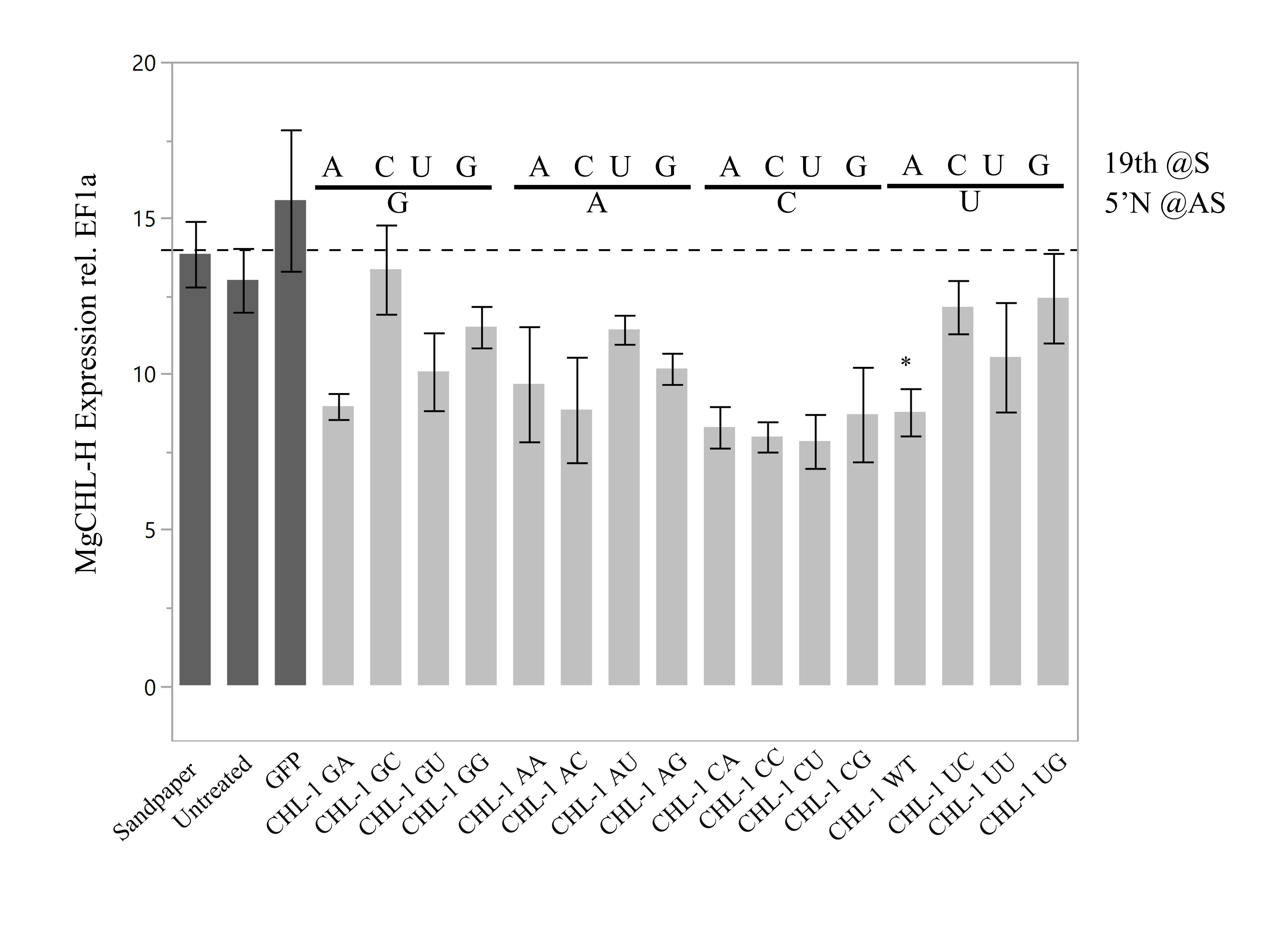

Supplement: S3 Fig — CHLH mRNA levels were determined in leaves harvested 4 dpa; error bars represent standard error of the means. The asterisk denotes the treatment of the wildtype siRNA that starts with a 5’U on the antisense strand with a paired A on the sense strand. (TIF) [file pone.0256863.s003.tif]

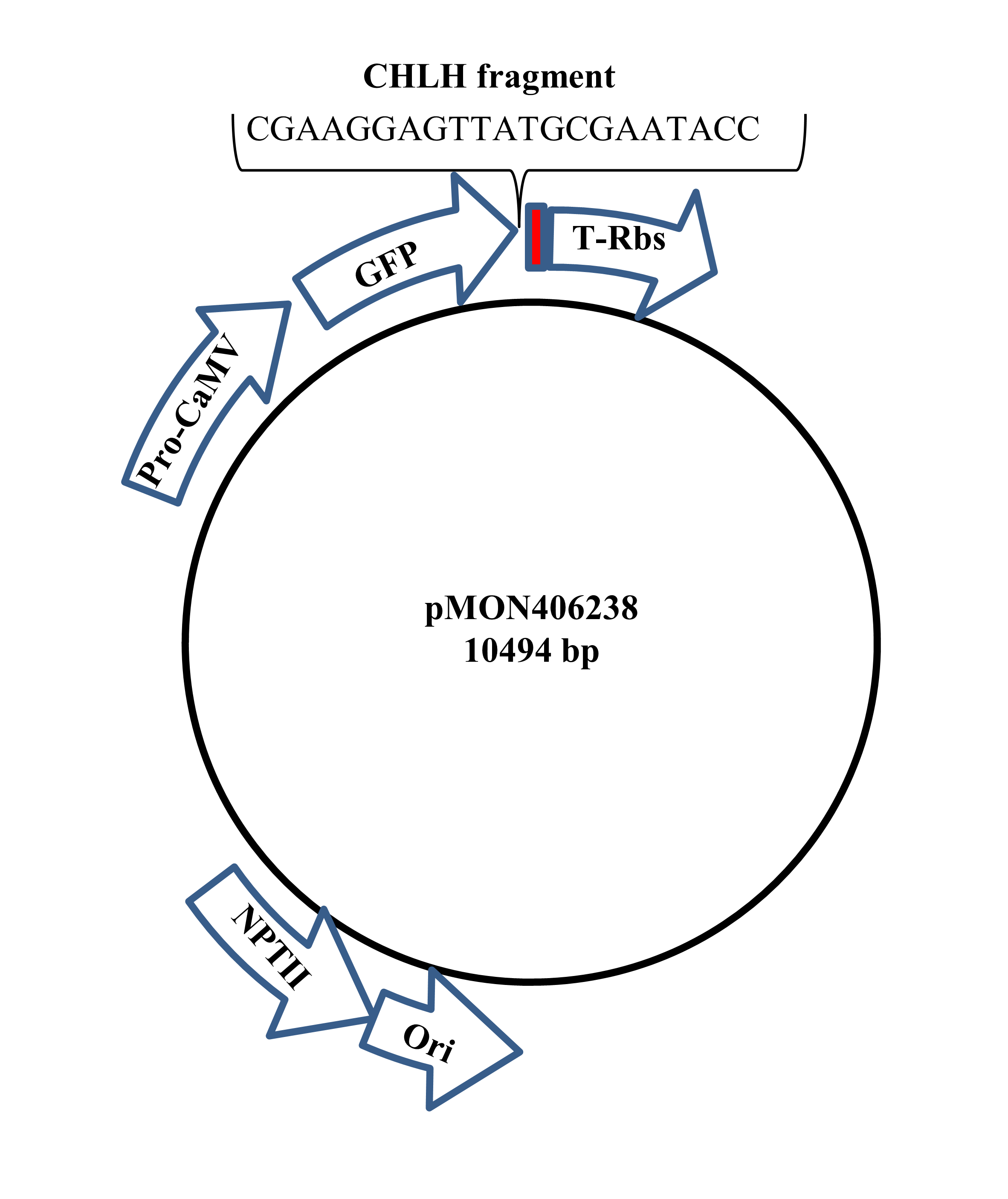

Supplement: S4 Fig — The GFP sequence is followed by the short stretch of CHLH sequence (5’-CGAAGGAGTTATGCGAATACC-3’). The expression of GFP is driven by the CaMV promoter and the RbcS terminator. (TIF) [file pone.0256863.s004.tif]

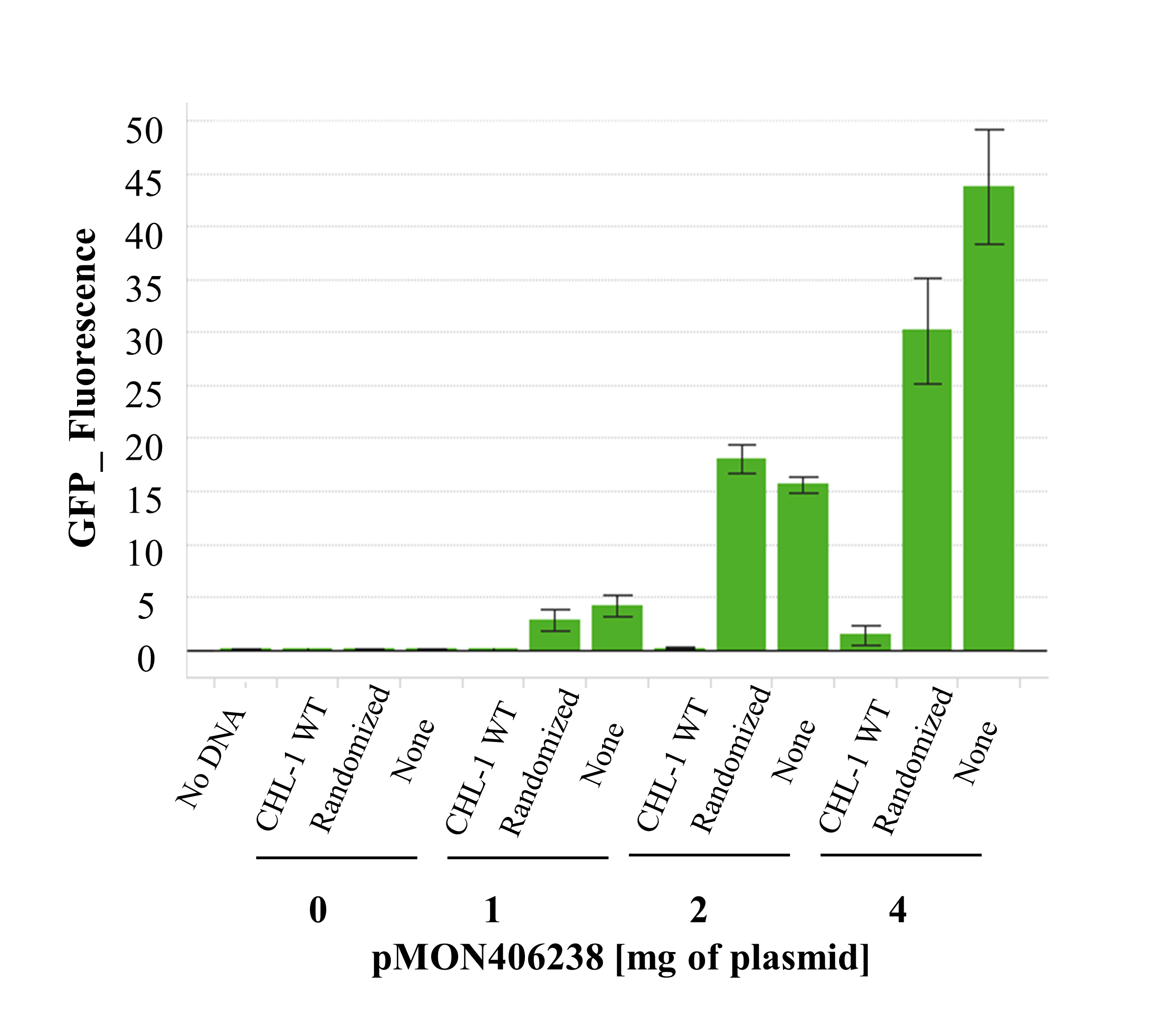

Supplement: S5 Fig — The reporter plasmid (pMON406238) was co-transfected at different concentrations into N. benthamiana protoplasts with 3 μg siRNA, and GFP fluorescence was determined after overnight incubation. RANDOMIZED, control siRNA. (TIF) [file pone.0256863.s005.tif]

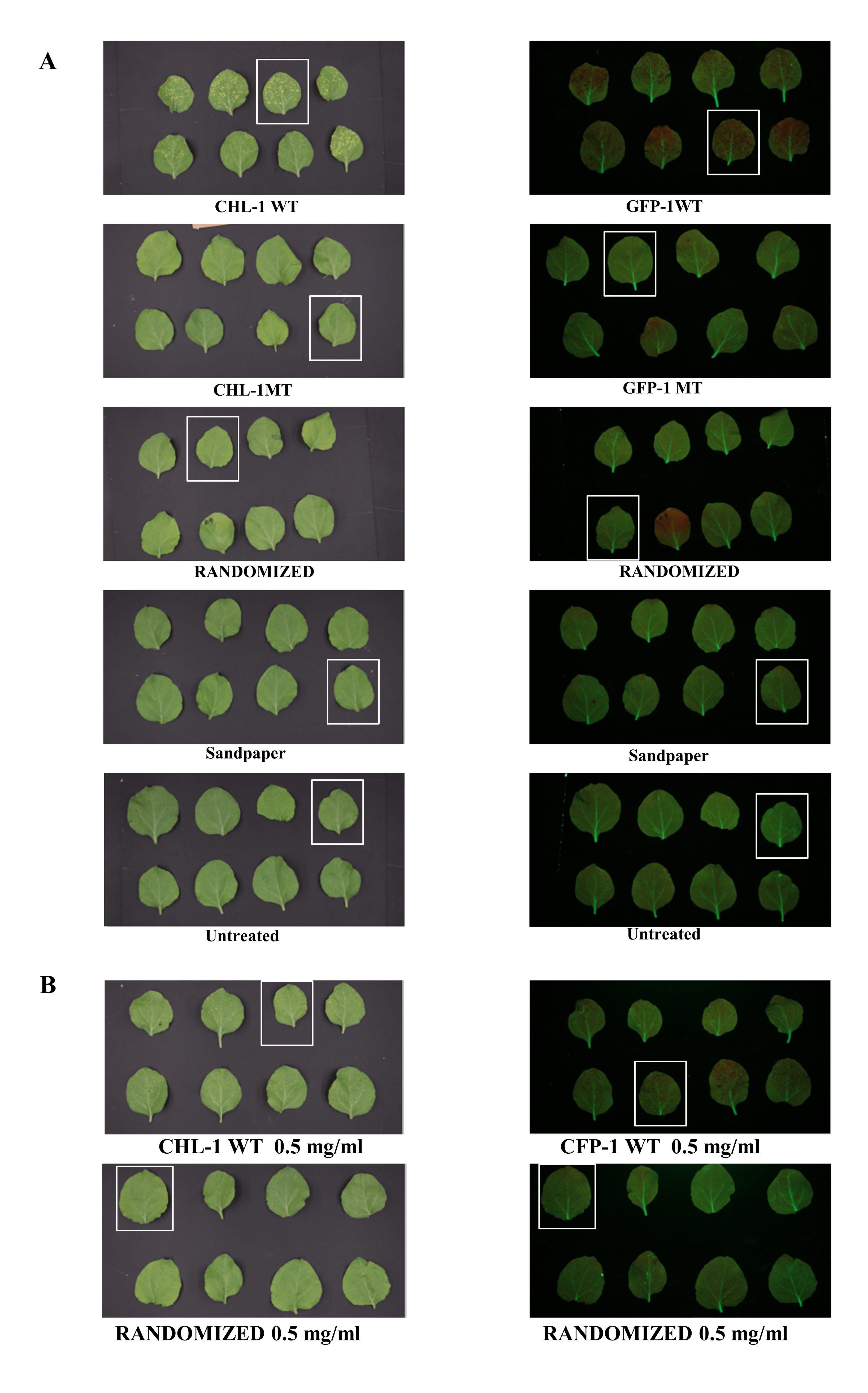

Supplement: S6 Fig — Leaves were treated with the indicated triggers by sandpaper abrasion and photographed 4 dpa to observe silencing phenotypes. White rectangle boxes highlight the leaf in each treatment shown in Fig 1(A) and S1(B) Fig. (TIF) [file pone.0256863.s006.tif]

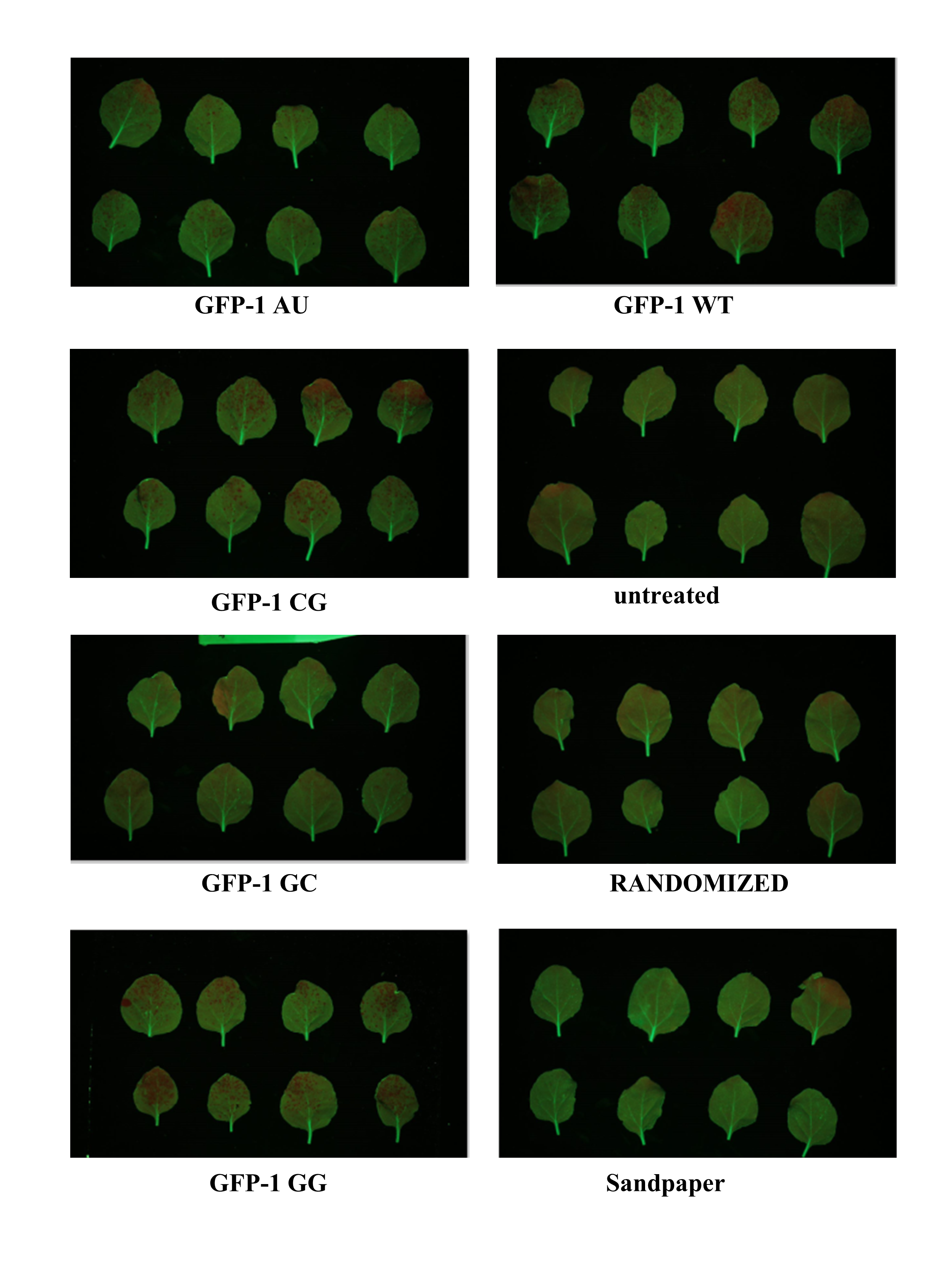

Supplement: S7 Fig — Leaves were treated with the indicated triggers by sandpaper abrasion and photographed under blue light 4 dpa to observe phenotypes. (TIF) [file pone.0256863.s007.tif]

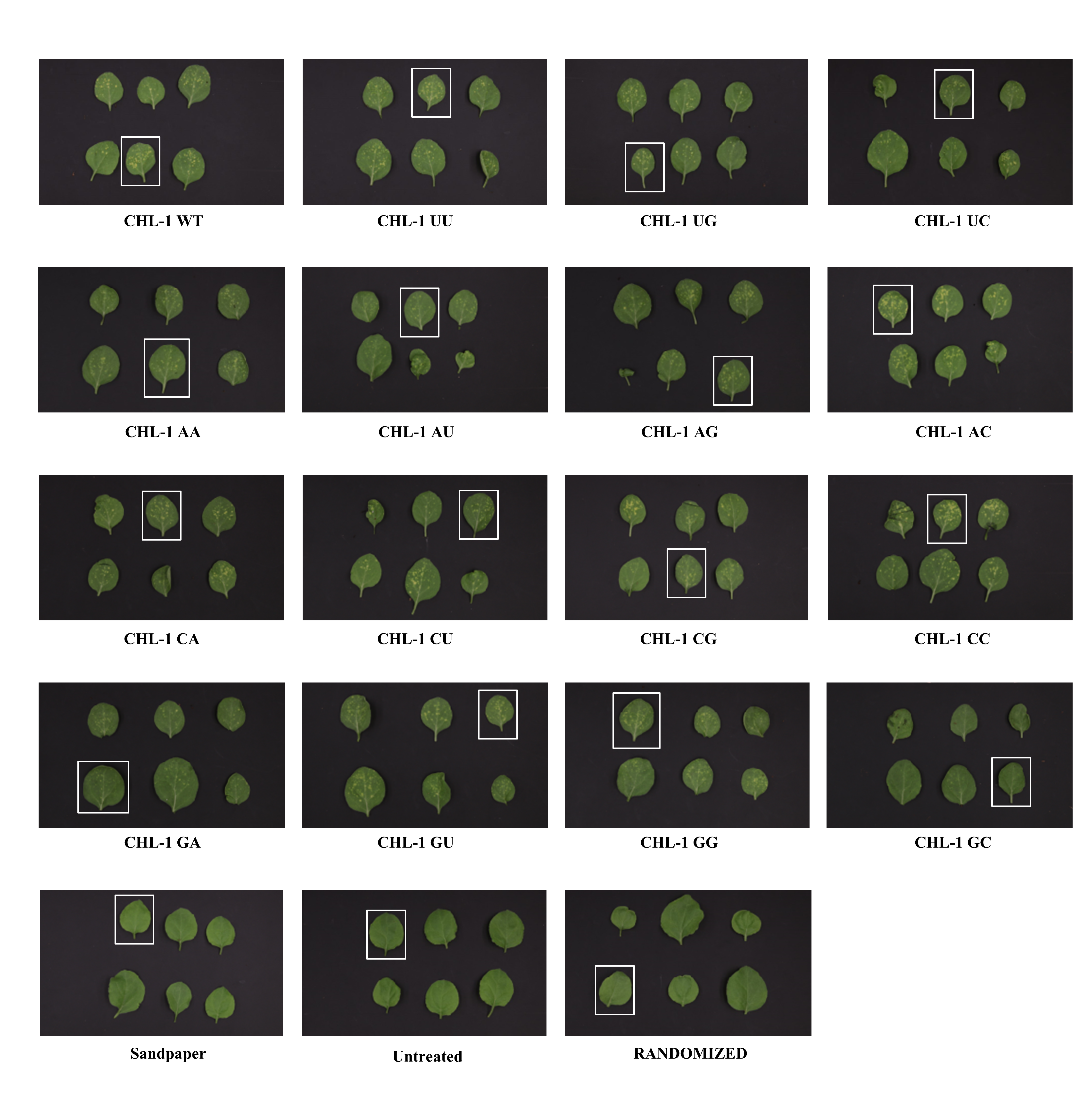

Supplement: S8 Fig — Leaves were treated with the indicated triggers by sandpaper abrasion and photographed under white light 4 dpa to observe silencing phenotypes. White rectangle boxes highlight the leaf in each treatment shown in Fig 3. (TIF) [file pone.0256863.s008.tif]

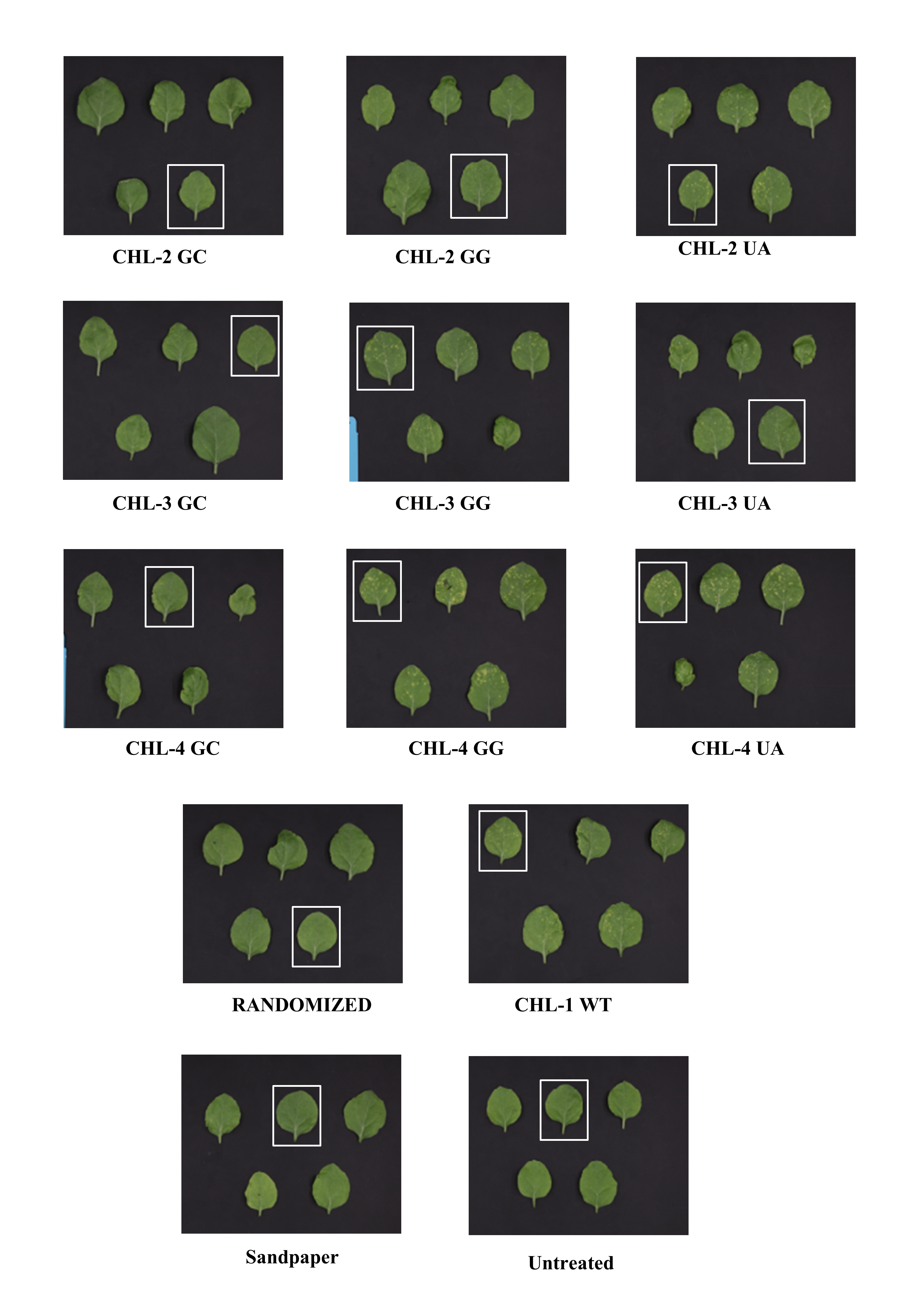

Supplement: S9 Fig — Leaves were treated with the indicated triggers by sandpaper abrasion and photographed under white light 4 days after treatment to observe silencing phenotypes. White rectangle boxes highlight the leaf in each treatment shown in Fig 5. (TIF) [file pone.0256863.s009.tif]
