# Supplementary material for: Beyond identity: Understanding the contribution of the 5’ nucleotide of the antisense strand to RNAi activity
Source: PLoS One. 2021 Sep 7;16(9):e0256863. doi: 10.1371/journal.pone.0256863 (PMC8423273; doi:10.1371/journal.pone.0256863)
Supplement: S1 Table — (DOCX) [file pone.0256863.s010.docx]

**S1 Table. RT-qPCR primer and probe sequences for transcript measurements (Sequences are written as 5’-3’)**

| GENE ID | TYPE | sequence |
| --- | --- | --- |
| *EF1a* | FWD Primer | CACGCATTGCTTGCTTCA |
|  | REV Primer | TCCATCTTGTTACAGCAGCAAATC |
|  | Probe | CTTGGTGTCAAGCAAA-MGB |
| *UKN1* | FWD Primer | ACTTGCTGAAGGTCTCTGTTG |
|  | REV Primer | AAGAGCTTTCCACCGATTCTC |
|  | Probe | TCTTTGGCCTTCCCCTACATTGATGC-TAMRA |
| *CHLH* | FWD Primer | CCAGGGCATTTTACGAGATGTG |
|  | REV Primer | TGCTGATATTGCTCCACGTGATG |
|  | Probe | SYBR DETECTION |
| *gfp* | FWD Primer | CAGGGGATCCCAACGAAAAGAGA |
|  | REV Primer | CATGTGTAATCCCAGCAGCTGTT |
|  | Probe | SYBR DETECTION |
