# Supplementary material for: Beyond identity: Understanding the contribution of the 5’ nucleotide of the antisense strand to RNAi activity
Source: PLoS One. 2021 Sep 7;16(9):e0256863. doi: 10.1371/journal.pone.0256863 (PMC8423273; doi:10.1371/journal.pone.0256863)

Fig. 1E Raw images of Ponceau staining of the total protein (top), and the western blot with anti-GFP antibody (bottom). Both images were captured by Bio-Rad imager, and exported as 600 dpi files.

Lane 1: Molecular weight marker , X indicates that is not included in the final figure
Lanes 2&3: 16C plants, treatments with GFP-1 WT siRNAs
Lanes 4&5: 16C plants, treatments with GFP-1 MT siRNAs
Lanes 6&7: 16C plants, treatments with randomized siRNAs
Lane 8: Non-transgenic WT Benthi plants, no treatment
Lane 9: 16C GFP plants, no treatment

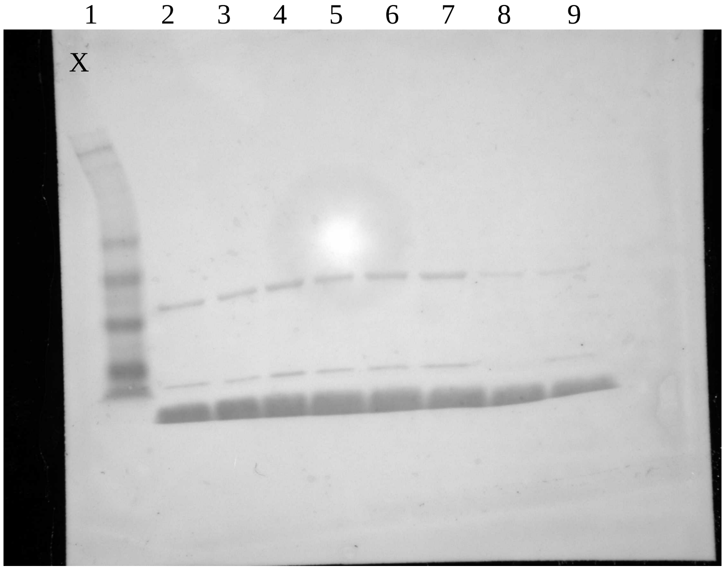

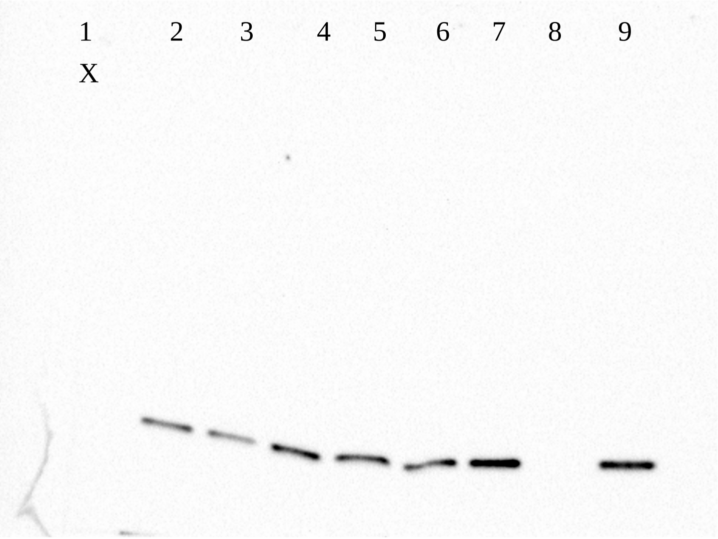

Supplement: S1 Raw images — (PDF) [file pone.0256863.s011.pdf]
