# Supplementary material for: Beyond identity: Understanding the contribution of the 5’ nucleotide of the antisense strand to RNAi activity
Source: PLoS One. 2021 Sep 7;16(9):e0256863. doi: 10.1371/journal.pone.0256863 (PMC8423273; doi:10.1371/journal.pone.0256863)
Supplement: Attachment — Submitted filename: Response to Reivewers_dataset link update.docx [file pone.0256863.s012.docx]

Bayer Crop Science
800 North Lindbergh Boulevard,

St. Louis, Missouri 63167,

United States of America

May 17th, 2021

Dear PLOS ONE Editors,

Here is an update on the data access from Dryad.

To access the original images/photos during the review period, please use this temporary link: <https://datadryad.org/stash/share/C1Xh6xhK3YeUfrVZJ5r86VIrJMscPHB_H_vpZJlLPaw>. The dataset has also been assigned a unique identifier, called a DOI (doi:10.5061/dryad.mcvdnck15), although this link will not be live until the dataset is published.

Data Availability: All the original images/photos to the dryad database with an accessible link as <https://doi.org/10.5061/dryad.mcvdnck15>. All other relevant data are located in the paper and its Supporting Information files.

We look forward to hearing from you regarding our re-submission. We would be glad to respond to any further questions and comments that you may have. Please address all correspondence concerning this manuscript to me at [peizhen.yang@bayer.com](mailto:peizhen.yang@bayer.com).

Sincerely,

Peizhen Yang, Ph.D.

Applied Cell Biology Team Lead

608-695-6167

Peizhen.yang@bayer.com
