# Supplementary material for: Beyond identity: Understanding the contribution of the 5’ nucleotide of the antisense strand to RNAi activity
Source: PLoS One. 2021 Sep 7;16(9):e0256863. doi: 10.1371/journal.pone.0256863 (PMC8423273; doi:10.1371/journal.pone.0256863)
Supplement: Attachment — Submitted filename: Response to Reivewers.docx [file pone.0256863.s013.docx]

Bayer Crop Science
800 North Lindbergh Boulevard,

St. Louis, Missouri 63167,

United States of America

August 7, 2021

Dear Dr. Sebastien Pfeffer,

Thank you for your leading this review process of our submission “***Beyond identity: understanding the contribution of the 5’ nucleotide of the antisense strand to RNAi activity***” to Plos One journal. We would like to sincerely thank both reviewers for their valuable time going through the revised manuscript and providing additional feedbacks. We appreciate reviewer 2’s recommendation of the revised version to the journal for publication, as well as reviewer 1’s acceptance of updates and the additional comments. Please see below our responses to reviewer 1’s comments (including the statistical calculations and terminal free energy, etc) and the corresponding changes made in the manuscript.

PONE-D-20-40091R1

Beyond identity: understanding the contribution of the 5’ nucleotide of the antisense strand to RNAi activity

Reviewer #1: The authors have now corrected several of the points I had raised in my initial review, but some still remain. Some of them really make the figures un-understandable (the usage of Student's t-test to "separate" (?) means; the undocumented usage of one-letter or two-letter codes to denote statistical significance). These points really need to be improved for the manuscript to be understandable.

Detailed remarks:

1. The new version of the manuscript now says that plant siRNAs bear 2´-O-methylation on their 3´ ends, which is good (lines 48-50), but the new formulation seems to imply that animal siRNAs always have a 3´ hydroxyl, which is not true at least in Drosophila. Please clarify.

The authors thank the reviewer for catching this. We updated the sentence to “siRNA duplexes are generally 20-25 nt in length, have 2nt 3’ overhangs and are bounded by 5’-phospate and 3’ hydroxyl moieties and additionally have 2’-O-methylation at their 3’ ends” to clarify the point that the siRNAs regardless of kingdom have 3’ hydroxyl and 2’ O- methylation by removing the “plant and animal” aspect of the original sentence.

2. The initial version of the manuscript was quantifying light intensity in micromoles. I suspected it was a mistake and asked for correction, and the new version now measures light intensity in "µmol m-2 s-1". Did the authors really get my point? Moles (and micromoles) measure amounts of particules (e.g., one mole of water is 6.02e23 molecules of water). Light intensity is measured in candelas, and light power is measured in watts. Not moles (or "moles per square meter and per second"). Unless the authors are counting moles of photons? Please clarify.

We did add the word “intensity” into our methods to clarify. The standard units for greenhouse lighting is measured in µmol m-2 s-1 or µmol/m2/s and for most if not all plant journals. We appreciate the attention to detail and can offer this explanation. The light intensity measurements are different for visible light for humans as opposed to greenhouse lighting. For human visible light, when describing light quantity or light intensity units - foot candles, lux , lumens etc are used. For greenhouse lighting, light intensity for plants is measured in a different range (400-700nm) and is referred to as Photosynthetic Active Radiation (PAR). The unit for measuring the PAR or instantaneous light incident upon a surface is micromoles per square meter per second (or µmol/m2/s) - this is the amount of energy (photons or particles of light) hitting a square meter every second. One source that explained this well was <http://www.greenhouse.cornell.edu/structures/factsheets/Greenhouse%20Lighting.pdf>. A scientifically published book chapter explaining PAR and a conversion table is <https://www.sciencedirect.com/topics/agricultural-and-biological-sciences/photosynthetically-active-radiation>. In plant science, researchers programming a greenhouse or growth chamber to recreate this study would very likely use a µmol/m2/s value to program the chamber or greenhouse settings.

3. The formulation "means were separated using Student's t-test" (lines 188, 190, 245, 304, 320 and 358) is extremely obscure. What did the authors exactly do? Student's t-test, just like the ANOVA, can assess whether the observed difference is significant (meaning that the sampled ideal populations have different means), except that Student's t-test can only compare 1 group to 1 group (one-way ANOVA can compare several groups to each other). So what has been "separated" here? Why has the Student's t-test been applied after the ANOVA?

There may be a misunderstanding of language around statistical analysis used in these studies. The reviewer is correct that one-way ANOVA compares means of several groups using the F-test statistic. A significant F-test indicates significant difference among all groups, a global test of significance, but does not provide specific test of the difference between any two groups. We evaluated the F-test and if the P-value was < 0.05, we then performed statistical tests of all possible pairwise comparisons using a t-test-. This method commonly referred to as Fischer’s-protected least significant difference. This is a classical method that provides protection for multiple testing and has been employed in across scientific disciplines for more than 40 years. This algorithm in implemented in the statistical software package we utilized (JMP12, SAS institute). We believe this method is appropriately used in these studies. We have altered the language in the text at each line pointed out by the reviewer to clarify this procedure.

4. The idea of indicating significant differences with a letter is a bit strange, it really doesn't help understanding. Why not simply adding asterisks on the bars with p-value < 0.05, as is classically done? The description of this counter-intuitive lettered code is obfuscated, making it hard for the reader to understand what is going on (see for example in the legend for Figure 1, line 246: "Means not followed by the same letter indicate statistical significance", but the figure does not indicate any mean value followed by any letter; I can only see letters "A" and "B" above the bars of the barplot, without any mean being written out). Figure 2B adds one more unexplained level of complexity, introducing 2-letter codes ("AB" and "BC") instead of 1-letter codes, and I cannot make sense out of it. The concept of displaying significance is quite simple, so please don't make it uselessly complicated with this undocumented letter code.

The use of letter codes to indicate significance is well-documented and commonly used in plant biology and many other disciplines. In the latest issue of PLOS One, Potcho et al (2021) use letter codes and Fishers protected LSD in the same way we utilized this common method on our studies. We revised the letter codes to lower case to meet PLOS One requirements, but we believe this statistical procedure is sound and supported by years of use in the literature, in statistical textbooks, and in the statistical software package used in this study (JMP12, SAS institute).

Potcho PM, Okpala NE, Korohou T, Imran M, Kamara N, et al. (2021) Nitrogen sources affected the biosynthesis of 2-acetyl-1-pyrroline, cooked rice elongation and amylose content in rice. PLOS ONE 16(7): e0254182. [https://doi.org/10.1371/journal.pone.0254182](%20https://doi.org/10.1371/journal.pone.0254182)

5. In response to my initial comments, the authors explained that the compared free energies at the duplex' extremities are not just stacking energies, but also base-pairing energies (which makes a lot of sense of course). But then why does the updated text still read (on lines 310-312) that the comparison was about "stacking energies of the first 5 pairs" and an obscure "free end loop"? What is this loop? Please be more rigorous on the terms (I thought I was clear in my first evaluation): stacking energy is just one term in the total pairing energy, and most likely the authors are comparing the total pairing energies here. Also, please detail what the "free en loop" is, and what is its role in this energy comparison (I really have no clue what this loop thing is).

The authors appreciate the feedback from the reviewer being more rigorous on the description of the “total pairing energy”.  Since the calculation of the delta G has been described in the earlier sentence in the same paragraph,  we have updated the sentence of  “as the differential free energy between two ends of the duplex of the first 5 pairs of the sequence and the free end loops.  Ct = 10uM, Na+ at 1M, and @ 37C.”  to “as the difference in thermodynamic stabilities at each end”.

Regarding the terminal stability (dG, kcal.mole-1) at the each end of the siRNA duplex, as described in the materials and methods section, we used the software of DINA melt  (<http://www.unafold.org/Dinamelt/applications/two-state-melting-hybridization.php>) by summing the first four nearest-neighbor energies and the free energy of 2nt overhang.  The free end loops refer to the 3’ 2nt overhangs that affect the free energy of the end of RNA structure.   In many studies comparing siRNA properties with large dataset, typically the last 2nt at 3’ end of both strands are set at “TT” so to cancel the energy contribution from the overhangs and simplify the calculation of ddG to just the 4 nearest neighbor energies from the stem region.  In this study,  we didn’t use the generic “TT” as the overhang but rather the actual matching sequences from the genes, so the terminal energy include the contribution of 2’ nt overhangs in addition to the four nearest-neighbor energies at the 5’ end of the siRNA.

In original publication by Turner and Matthews (see below) on the nearest-neighbour model, the overhang shown as below is described as an exterior loop (as is highlighted in the red circle). We borrowed this concept of “free-end loop“ to describe the overhang. We do agree with the reviewer that this description seems confusion, therefore we have updated in the text as overhang, to make the description clear.

Turner, D. H., & Mathews, D. H. (2010). NNDB: the nearest neighbor parameter database for predicting stability of nucleic acid secondary structure. *Nucleic acids research*, *38*(Database issue), D280–D282. <https://doi.org/10.1093/nar/gkp892>

Reviewer #2: In this revised manuscript, Yang and colleagues investigated whether 5’-nt identity is an important determinant of posttranscriptional gene silencing triggered by topically-applied siRNAs in plants. They used sandpaper abrasion to introduce synthetic 21 bp siRNA duplexes to N. benthamiana leaves, then evaluated silencing of genes homologous to those siRNAs. Deficiency for an endogenous target in N. benthamiana, CHLH, produces white spots. Silencing of a GFP transgene, on the other hand, reduces GFP fluorescence in N. benthamiana leaves, leading to red fluorescent spots under blue light illumination (concerning this revision, I thank the authors for correcting my mistake about the GFP5 excitation wavelength). The authors measured this gene silencing by detecting GFP mRNA levels (RT-qPCR) and GFP protein (western blot).

In their revised manuscript the authors addressed all the minor issues that I raised, and several substantive concerns of the other referee. They now cite the missing studies, have better annotated figures and have explained the other technical aspects that were confusing in the original manuscript.

The authors present rigorous findings on how siRNA duplex thermostability influences the efficiency of RNA silencing by exogenous siRNAs. Understanding this process has implications for crop protection and weed control. This manuscript will be of interest to the field of RNA silencing in plants, particularly to researchers looking for advice on applications involving siRNA delivery.

We look forward to hearing from you regarding our re-submission. We would be glad to respond to any further questions and comments that you may have. Please address all correspondence concerning this manuscript to me at [peizhen.yang@bayer.com](mailto:peizhen.yang@bayer.com).

Sincerely,

Peizhen Yang, Ph.D.

Applied Cell Biology Team Lead

608-695-6167

Peizhen.yang@bayer.com
